# A Semi-Supervised Approach to Bearing Fault Diagnosis under Variable Conditions towards Imbalanced Unlabeled Data

**DOI:** 10.3390/s18072097

**Published:** 2018-06-29

**Authors:** Xinan Chen, Zhipeng Wang, Zhe Zhang, Limin Jia, Yong Qin

**Affiliations:** 1 State Key Lab of Rail Traffic Control and Safety, Beijing Jiaotong University, Beijing 100044, China; 15114217@bjtu.edu.cn (X.C.); zhangzhe@bjtu.edu.cn (Z.Z.); yqin@ bjtu.edu.cn (Y.Q.); 2 National Engineering Laboratory for System Safety and Operation Assurance of Urban Rail Transit, Guangzhou 510000, China; 3 Beijing Research Center of Urban Traffic Information Sensing and Service Technologies, Beijing Jiaotong University, Beijing 100044, China

**Keywords:** rolling element bearing, semi-supervised learning, visibility graph, imbalanced data

## Abstract

Fault diagnosis of rolling element bearings is an effective technology to ensure the steadiness of rotating machineries. Most of the existing fault diagnosis algorithms are supervised methods and generally require sufficient labeled data for training. However, the acquisition of labeled samples is often laborious and costly in practice, whereas there are abundant unlabeled samples which also imply health information of bearings. Thus, it is worthwhile to develop semi-supervised methods of fault diagnosis to make effective use of the plentiful unlabeled samples. Nevertheless, considering the normal data are much more than the faulty ones, the problem of imbalanced data exists among unlabeled samples for fault diagnosis. Besides, in practice, bearings often work under uncertain and variable operation conditions, which would also have negative influence on fault diagnosis. To solve these issues, a novel hybrid method for bearing fault diagnosis is proposed in this paper: (1) Inspired by visibility graph, a novel fault feature extraction method named visibility graph feature (VGF) is proposed. The obtained features by VGF are natively insensitive to variable conditions, which has been validated by a simulation experiment in this paper; (2) On basis of VGF, to deal with imbalanced unlabeled data, graph-based rebalance semi-supervised learning (GRSSL) for fault diagnosis is proposed. In GRSSL, a graph based on a weighted sparse adjacency matrix is constructed by the k-nearest neighbors and Gaussian Kernel weighting algorithm by means of the samples. Then, a bivariate cost function over classification and normalized label variable is built up to rebalance the importance of labels. Finally, the proposed VGF-GRSSL method was verified by data collected from Case Western Reserve University Bearing Data Center. The experiment results show that the proposed method of bearing fault diagnosis performs effectively to deal with the imbalanced unlabeled data under variable conditions.

## 1. Introduction

Rolling element bearings are one of the most frequently used components in machines. Reference [1] shows that a large part of faults in machines (above 40% of the total faults) owes to bearings. Therefore, bearing fault diagnosis has been a research hotspot for decades. Reference [2] reviewed the diagnostic techniques over the past decade and summarized six trends in fault diagnosis for the electrical machines. So far, many studies have proposed plenty of feature extraction methods, such as Fourier transform, wavelet transform, empirical mode decomposition, fuzzy entropy et al. [3,4,5,6]. Besides, machine learning methods, including support vector machine, decision tree, neural network [7,8,9], are utilized to classify various bearing fault categories and achieve high accuracy rates of fault diagnosis. In addition, many studies have used novel observation of the bearing to detect incipient phase bearing faults, such as those based on current signals and stray flux measurement in different positions [10,11].

However, although the aforementioned algorithms perform well, reference [12] indicates that the classifications based on supervised learnings tend to perform poorly due to inadequate labeled training data. It is because the classifiers are supposed to remember the training samples instead of learning rules from them and easily lead to overfitting. However, this issue is rarely discussed in the field of mechanical fault diagnosis. In practice, it is laborious or expensive to collect faulty samples with labels, whereas the unlabeled samples are abundant [13,14]. Therefore, it is valuable to develop semi-supervised methods for fault diagnosis to improve the accuracy as much as possible by means of a few labeled data and mass unlabeled data.

Meanwhile, the monitoring data of bearings are usually collected during runtime. Since most of the time, bearings work under normal conditions, normal samples acquired from bearings are much more than the faulty ones. Therefore, the distributions of bearing data are seriously imbalanced in practice [15]. Therefore, the semi-supervised approach to bearing fault diagnosis suffers from the problem of imbalanced data. Besides, in the real world, bearings often work under variable and fluctuant conditions. The labeled and unlabeled data are gathered from bearings with speed variations in practice. It is essential to study feature extraction under variable conditions. Therefore, to deal with these aforementioned issues, this paper mainly discusses how to develop a highly-accurate diagnosis for bearings under variable conditions with imbalanced unlabeled data.

To this end, a novel hybrid method for bearing fault diagnosis is presented in this paper. Inspired by visibility graph, a novel fault feature extraction method named visibility graph feature (VGF) is proposed to extract fault characteristics under variable conditions. The VGF method coverts vibration signals into graphs and extracts features based on structures of these graphs. Since the visibility remains invariant under horizontal and vertical transformation of time series, the obtained VGFs are natively insensitive to variable conditions, which is verified in the following simulation experiments. Based on the VGFs, graph-based rebalance semi-supervised learning (GRSSL) is employed for fault classification. As a new semi-supervised method, GRSSL [16] is proposed by in. In GRSSL, a graph is established from data based on a kernel function, and then the k-nearest neighbors (KNN) and Gaussian Kernel weighting algorithm are employed to sparsify and reweight the connections. During the optimization process, a bivariate function over classification function and the unknown labels is built up, and then, a normalized label variable is used to rebalance the importance of labels. In this paper, experiments are designed to test the performance of the proposed GRSSL and VGF. The results have shown that the GRSSL outperforms the popular methods, including Gaussian Fields and Harmonic Functions (GFHF) [17] and Local and Global Consistency (LGC) [18].

The rest of the paper is organized as follows. Section 2 reviews the related literature. The novel VGF and GRSSL are presented in Section 3. Section 4 describes the simulation experiment for VGF. Experiments are analyzed in detail in Section 5, and Section 6 presents the key conclusions.

## 2. Related Work

Feature extraction is a key step for bearing fault diagnosis. There have been many studies on feature extraction under variable conditions. Borghesani et al. [19] proposed a new procedure for using envelop analysis to remove the effects of variable conditions, but required prior knowledge about defect frequencies of bearings. Feng et al. [20] exploited a concentration of frequency and time method to deal with the variable speed conditions, but required prior information about the undergoing conditions. Tian et al. [21] employed local mean decomposition method and extreme learning machine to detect the bearing fault under variable operation conditions, but required complete condition data. Wang et al. [22] conjugated variation mode decomposition and singular value decomposition to extract features which can fit the variable conditions adaptively. However, it is even impossible for such prior knowledge and complete data for all conditions. Therefore, it is essential to extract fault features which are natively insensitive to variable conditions. Visibility graph(VG), proposed by Lacasa et al. [23] intends to convert time series into complex networks, and the obtained structure parameters not only imply the characteristics of the raw data, but also keep invariant under several transformations of the data. Therefore, inspired by VG, a novel feature extraction method under variable conditions is proposed in this paper.

Fault classification is the following step after feature extraction. There are a large number of references on vibration-based bearing fault classification [7,8,9]. However, most of the existing methods purely depend on labeled data for training. The masses of unlabeled samples acquired in practice are abandoned. To make effective use of those valuable unlabeled samples, the semi-supervised learning (SSL) [24], which is capable of exploiting the unlabeled data combined with a small amount of labeled data to train a well-performed classifier, has been a highlight issue in the field of bearing fault diagnosis.

Concerning this issue, Li [25] proposed a semi-supervised weighted kernel clustering algorithm based on gravitational search for bearing fault diagnosis and processed the unlabeled samples by calculating the weighted kernel distances among them and fault cluster centers. Qin et al. [26] employed a tritraining method for bearing fault diagnosis. Zhao et al. [27] proposed a new graph-based semi-supervised classification for bearing fault diagnosis with sparse coding method. However, these existing semi-supervised methods of bearing fault diagnosis have not considered the imbalance of the unlabeled data. Therefore, it is crucial to develop novel semi-supervised methods for bearing fault diagnosis to deal with the imbalanced unlabeled data.

Among the semi-supervised methods, Graph-based semi-supervised learning (GSSL) [17,18] considers samples as vertices in a graph and shapes the pairwise edges, which are determined by the similarity between the corresponding samples. Then, the small portion of labels propagate to predict labels for unlabeled samples by semi-supervised learning method. Subramanya et al. [28] have proved that GSSL outperforms non-graph-based SSL approaches. Based on the GSSL, Jebara et al. [16] extends a bivariate optimization by which both classification function and a normalized label variable are considered to rebalance the importance of labels. Therefore, the graph-based rebalance semi-supervised learning (GRSSL) is employed for fault classification to deal with the imbalanced unlabeled data.

The intended contributions of this study can be summarized as follows:

1. A novel fault feature extraction method named visibility graph feature (VGF) is proposed to extract fault characteristics under variable conditions. The vibration signals are mapped into graphs whose structures are natively insensitive to variations of signals corresponding to the speed and load conditions.
2. A GRSSL algorithm by blending the normalized label variable is developed for bearing fault diagnosis with imbalanced and unlabeled data. To cope with the imbalanced problem, a bivariate optimization function is employed, and the importance of labels is rebalanced by the normalized label variable term.

## 3. Methodology

**Figure 1:**
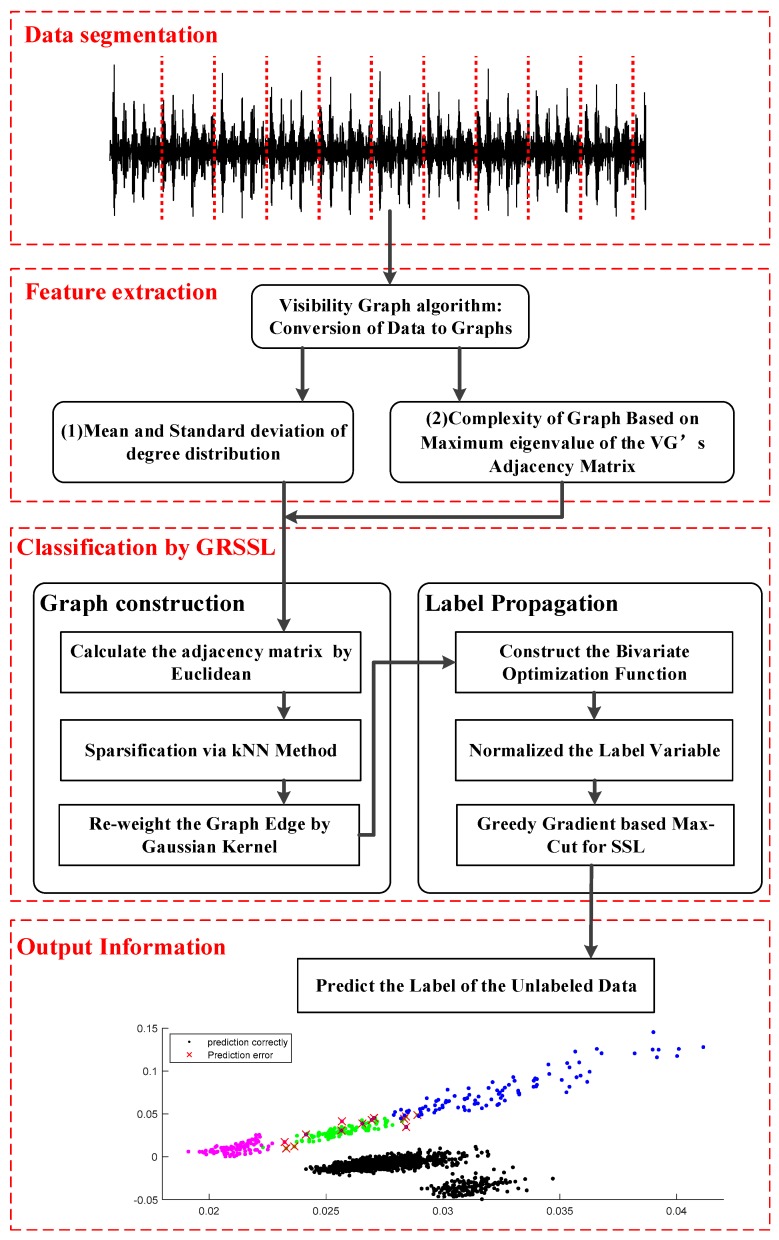
The overall scheme of proposed fault diagnosis method.

In this section, the proposed fault diagnosis method based on VGF-GRSSL is described in detail, as shown in Figure 1. There are four layers in the method: data segmentation layer, feature extraction layer, fault classification layer, and output layer.

1. Data segmentation layer: The signals are segmented according to the sample rate and rough shaft speed to ensure each obtained sample covers several circles of signals.
2. Feature extraction layer: The samples are mapped into visibility graphs. Then, features based on the structures of graphs including degree distribution of visibility graph (DDVG) and graph index complexity (GIC) are extracted.
3. Fault classification layer: This layer includes graph construction and label propagation.

In the graph construction, an adjacency matrix is calculated based on a kernel function firstly, and then sparsified and reweighted by means of the k-nearest neighbors (KNN) and Gaussian Kernel weighting algorithm, respectively. Then, a graph with a weighted sparse undirected adjacency matrix is calculated from the samples.

During the label propagation process, a bivariate function over classification function and the unknown labels is built up, and then, a normalized label variable is used to rebalance the importance of labels. Finally, the greedy gradient-based Max-Cut algorithm is adopted to solve the optimization model.

4. Output layer: The unlabeled data is marked with the corresponding label according to the classification with few labeled data. The GRSSL classification based on VGF is expected to extend the bearing fault diagnosis under variable condition with imbalanced unlabeled data.

### 3.1. Visibility Graph Feature Extraction

#### 3.1.1. Construction of Visibility Graph

The theory of visibility graph has been lucubrated for years and applied widely on many fields, such as engineering and urban planning [29]. Lacasa et al. [23] introduced the theory to the analysis of time series. Here, an undirected complex network is established by individual observations in a time series whose connectivity is defined through the visibility condition in physical space. It has been proved that the obtained graph inherits the intrinsic properties of the raw time series. For instance, the values of impulses of time series are related to the hubs of the graph, and the periods of time series are related to the degree distributions of the graphs. Motivated by this creative idea, this paper proposes a novel feature extraction method based on visibility graph.

**Figure 2:**
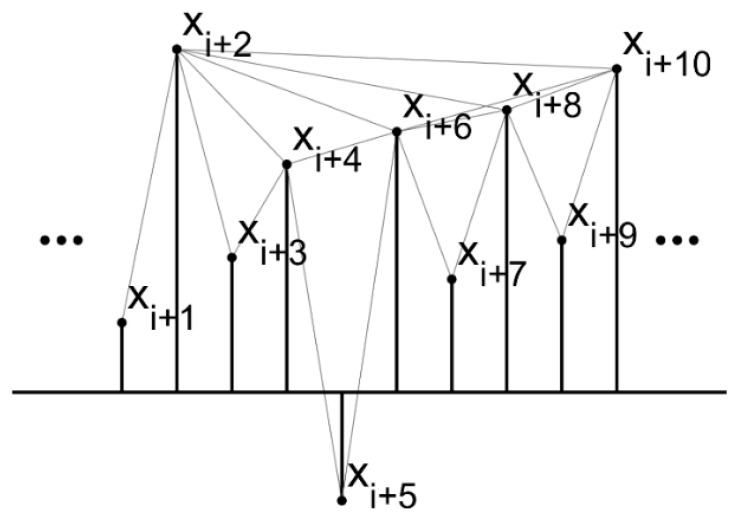
Illustration of converting a time series into a visibility graph.

An example is given to describe how to convert a time series into a visibility graph, as shown in Figure 2. There are 10 points of a random series. Then, the visibility exists between any two points if there is a straight line that does not intersect any intermediate data height. The complexity of the time series is supposed to be revealed in the structure of the obtained visibility graph.

The visibility criteria can be formulated as follows: two arbitrary points $\left(t_{a},x_{a}\right)$ and $\left(t_{b},x_{b}\right)$ of the series data will be connected as nodes of the graph, if the points $\left(t_{c},x_{c}\right)$ between them fufill:

(1) $$x_{c}<x_{b}+(x_{a}-x_{b})\frac{t_{b}-t_{c}}{t_{b}-t_{a}}$$

**Figure 3:**
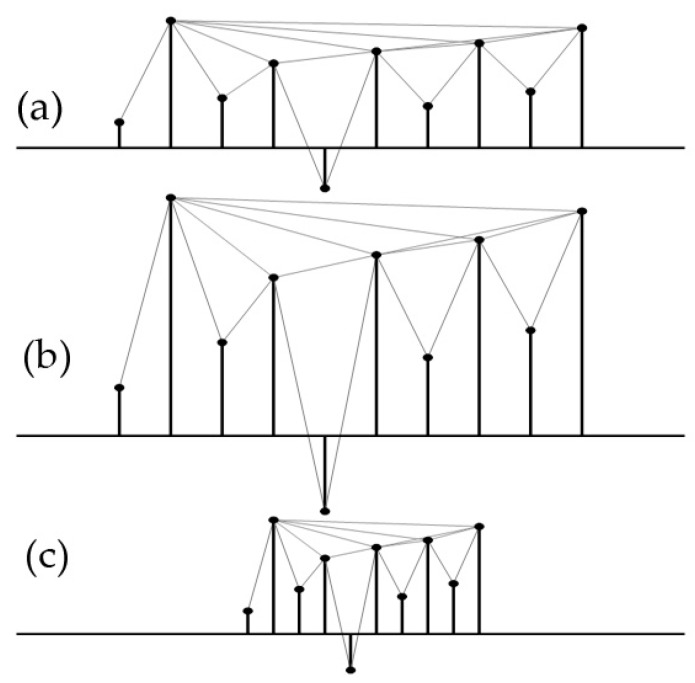
The visibility remains invariant under horizontal and vertical transformation of the time series. (**a**) Visibility links of the original time series; (**b**) Resizing vertically; (**c**) Resizing horizontally.

It is obviously that the associated graph constructed from a time series is always:

1. Connected: each node can see its nearest two neighbors at least.
2. It is an undirected link between every two node.
3. Invariant under affine transformations: the visibility keeps invariant under the resizing of both horizontal and vertical axes (as shown in Figure 3). Therefore, the visibility-graph-based features are natively insensitive to variable conditions.

#### 3.1.2. Features Based on Visibility Graph

After the construction of the graph, an adjacent matrix $W_{V}\in B^{n\times n}$ can be defined to record the links of the graph. It is a symmetric matrix, and the values of elements are either 1 or 0, which indicate that there is a connection or not between the corresponding nodes. For example, if one of the elements $w_{ij}=1$, it indicates that the node i and node j are connected with each other. Then, degree distribution of visibility graph (DDVG) and graph index complexity (GIC) are involved for feature extraction under variable conditions.

##### Degree Distribution of Visibility Graph (DDVG)

Define the degree distribution $D_{V}=\left[d_{1},\cdots ,d_{n}\right]$ with $d_{i}=\sum_{j=1}^{n}w_{ij}$, which indicate the connection situation of the graph. Then, the mean and standard deviation of the degree distribution ($Mean\left(D_{v}\right)$ and $\mathrm{Std}\left(D_{v}\right)$) are calculated as fault features.

**Figure 4:**
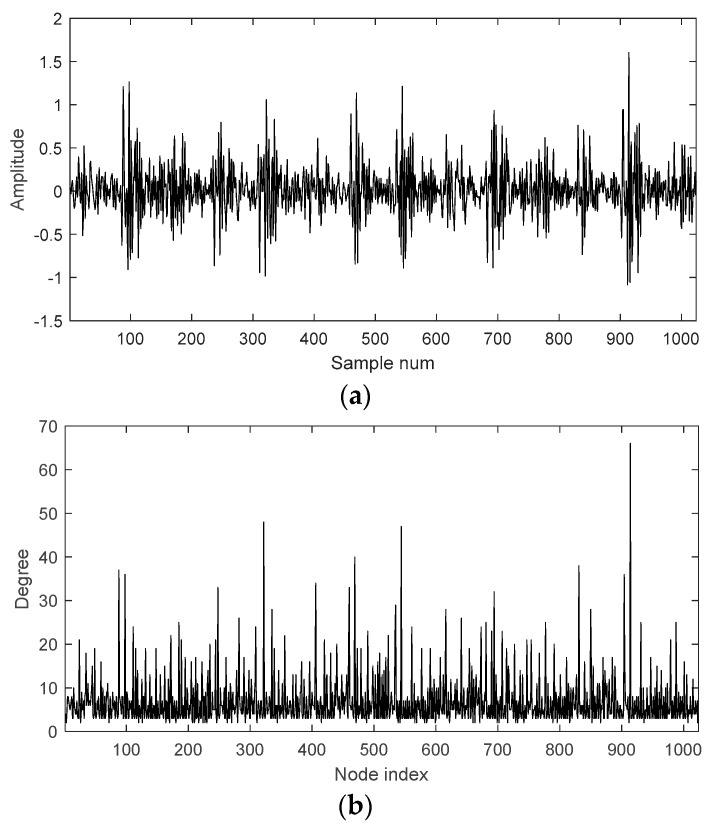
(**a**) A sample of faulty data and (**b**) degree distribution of its VG.

Figure 4a shows a sample of faulty data with 1024 points. Figure 4b shows the degree distribution of its VG with 1024 nodes. There are several nodes with large values in the degree distribution. Such nodes are hubs of the graph and imply the corresponding points in the time series have large or small values.

##### Graph Index Complexity (GIC)

As a measure of complexity of a graph, GIC [30] is also involved for feature extraction in this study. GIC is defined as follows:

(2) C=4c(1−c)

where

(3) $$c=\frac{\lambda_{\max}-2\cos\left(\pi /\left(n+1\right)\right)}{n-1-2\cos\left(\pi /\left(n+1\right)\right)}$$

$\lambda_{\max}$ represents the largest eigenvalue of the adjacency matrix of a graph with n nodes.

### 3.2. Fault Classification Based on GRSSL

Assume that there are labeled samples $\left\{\left(x_{1},y_{1}\right),\cdots ,\left(x_{l},y_{l}\right)\right\}$ and unlabeled samples $\left\{x_{l+1},\cdots ,x_{l+u}\right\}$ derived from an identical independent distribution. Define $X_{l}=\left\{x_{1},\cdots ,x_{l}\right\}$ and $X_{u}=\left\{x_{l+1},\cdots ,x_{l+u}\right\}$ as the set of labeled inputs and unlabeled inputs, respectively. l and u represent the number of labeled and unlabeled samples. $Y=\left\{y_{ij}\right\}\in B^{n\times c}$ is the label information matrix, where $y_{ij}=1$ if the sample *x_i_* is related to label j, j∈{1,2,⋯,c}. Each sample can be marked with a label: $\sum_{j=1}^{c}y_{ij}=1$. The semi-supervised learning aims to identify the missing labels $\left\{y_{l+1},\cdots ,y_{l+u}\right\}$ of unlabeled samples, where typically l<<n(l+u=n); there are two parts of the graph-based rebalance semi-supervised learning (GRSSL): graph construction and label propagation.

#### 3.2.1. Graph Construction

In this paper, assume the G={X,E,W} represents the undirected graph from the data ${X=X}_{l}\cup X_{u}$. There are usually two steps in the estimation of G from X.

1. The sample similarities are calculated by using a kernel function and utilized to construct a full adjacency matrix K: $K\in R^{n\times n},K_{ij}=k\left(x_{i},x_{j}\right)$
2. The matrix K is c and reweighted to obtain the final matrix W.

The sparsification deletes edges of the matrix K by multiplying a binary matrix $B\in B^{n\times n}$ and a distance matrix $H\in R^{n\times n}$. The binary and distance matrix are defined as follows:

(4) $$B_{ij}=\{\begin{array}{cc} 1 & x_{i}\;connect\;x_{j}\\ 0 & x_{i}\;disconnect\;x_{j} \end{array}$$

(5) $$H_{ij}=\sqrt{K_{ii}+K_{jj}-2K_{ij}}$$

In this study, the *k*-nearest neighbor algorithm is employed to estimate the binary matrix *B*. The optimization procedure can be defined as follows:

(6) $$\begin{array}{l} \underset{\hat{B}\in B}{\min}\sum_{ij}{\hat{B}}_{ij}H_{ij}\\ s.t.\sum_{j}{\hat{B}}_{ij}=k,{\hat{B}}_{ii}=0,\forall i,j\in 1,\ldots ,n. \end{array}$$

The final binary matrix is computed by:

(7) $$B_{ij}=\max\left({\hat{B}}_{ij},{\hat{B}}_{ji}\right)$$

After the construction of the sparsified graph, the Gaussian Kernel Weighting algorithm is employed to calculate the weight matrix W. The weight between $x_{i}$ and $x_{j}$ is computed as follows:

(8) $$w_{ij}=B_{ij}\exp\left(-\frac{d^{2}\left(x_{i},x_{j}\right)}{2\sigma^{2}}\right)$$

where, $d\left(x_{i},x_{j}\right)$ represent the Euclidean distance of $x_{i}$ and $x_{j}$.

Finally, a graph G with a weighted sparse adjacency matrix W is generated from the data. 

#### 3.2.2. Label Propagation

Given the constructed graph G={X,E,W}, the purpose of label propagation is to diffuse the known labels to all unlabeled nodes $X_{u}$ in the graph and infer $Y_{u}$. In this study, this issue is cast as a bivariate optimization over the classification function F and the unknown labels Y:

(9) $$\begin{array}{l} \left(F^{*},Y^{*}\right)=\arg\;\min_{F\in R^{n\times c},Y\in B^{n\times c}}Q\left(F,Y\right)\\ \;=\arg\;\min_{F\in R^{n\times c},Y\in B^{n\times c}}\left(Q_{smooth}\left(F\right)+Q_{fit}\left(F,Y\right)\right)\\ \;s.t.\;y_{ij}\in \{0,1\},\\ \;\sum_{j=1}^{c}y_{ij}=1,j=1,\cdots ,c.\\ \;y_{ij}=1,for\;\mathrm{label}\left(x_{i}\right)=j,j=1,\cdots ,c. \end{array}$$

where Y is a binary matrix. The constraint $\sum_{j=1}^{c}y_{ij}=1$ indicates that it is a single label prediction issue, and $\left(F^{*},Y^{*}\right)$ represents the optimal solution. The cost function can be specified as:

(10) $$\begin{array}{cc} Q\left(F,Y\right) & ={\left\|F\right\|}_{G}^{2}+\frac{\mu }{2}{\left\|F-Y\right\|}^{2}\\ & =\frac{1}{2}\mathrm{tr}\left(F^{T}LF+\mu {\left(F-Y\right)}^{T}\left(F-Y\right)\right) \end{array}$$

where the first item ranks the function smoothness [31] over graph G, and the second item represents the loss to fit label matrix. The normalized graph Laplacian are as follows:

(11) $${L=D}^{-1/2}\left(D-W\right)D^{-1/2}=I-D^{-1/2}WD^{-1/2}$$

where the vertex degree matrix $D=diag\left(\left[d_{1},\cdots ,d_{n}\right]\right)$ with $d_{i}=\sum_{j=1}^{n}w_{ij}$.

The cost function can be rewritten as follows:

(12) $$Q\left(F,Y\right)=\frac{1}{2}\sum_{i=1}^{n}\sum_{j=1}^{n}w_{ij}{\left\|\frac{F_{i\cdot }}{\sqrt{d_{i}}}-\frac{F_{j\cdot }}{\sqrt{d_{j}}}\right\|}^{2}+\frac{\mu }{2}\sum_{i=1}^{n}{\left\|F_{i\cdot }-Y_{i\cdot }\right\|}^{2}$$

where $F_{i\cdot }$ denote the *i*’th row vectors of F.

The bivariate issue is converted to a univariate issue in regards to the label variable Y.

(1)   Optimization of F:

F is continuous, and the issue is convex while Y is fixed. The minimum is easy to be calculated by setting the partial derivative to zero:

(13) $$\begin{array}{l} \frac{\partial Q}{\partial F^{*}}=0\Rightarrow LF^{*}+\mu \left(F^{*}-Y\right)=0\\ \Rightarrow F^{*}={\left(L/\mu +I\right)}^{-1}Y=PY \end{array}$$

where, $P={\left(L/\mu +I\right)}^{-1}$ is defined as the propagation matrix.

(2)   Optimization of Y:

The optimal $F^{*}$ is employed in Equation (10) instead of F.

(14) $$\begin{array}{cc} Q\left(Y\right) & =\frac{1}{2}\mathrm{tr}\left(Y^{T}P^{T}LYP+\mu {\left(PY-Y\right)}^{T}\left(PY-Y\right)\right)\\ & =\frac{1}{2}\mathrm{tr}\left(Y^{T}\left(P^{T}LP+\mu \left(P^{T}-I\right)\left(P-I\right)\right)Y\right)=\frac{1}{2}\mathrm{tr}\left(Y^{T}AY\right) \end{array}$$

where, $A=P^{T}LP+\mu \left(P^{T}-I\right)\left(P-I\right)=P^{T}LP+\mu {\left(P-I\right)}^{2}$.

To cope with the imbalanced data, a normalized label variable $\tilde{Y}=\Lambda Y$ is utilized to replace the original Y. The diagonal matrix $\Lambda =\mathrm{diag}\left(\left[\lambda_{1},\cdots ,\lambda_{n}\right]\right)$ is imported to rebalance the importance of labels based on node degrees. In the formula (15), the degree of the vertex represents its importance in the graph. The majority class is supposed to correspond to a larger $\sum_{k}y_{kj}d_{k}$ than that of the minority class, so the sample from majority has a small $\lambda_{i}$ that can reduce its importance in the graph. The sum of $\lambda_{i}$ of each class is equal to 1. That means that they shared the same importance in the graph. Therefore, the diagonal matrix is expected to eliminate the influence of the imbalanced data.

(15) $$\lambda_{i}=\{\begin{array}{cc} p_{j}\cdot \frac{d_{i}}{\sum_{k}y_{kj}d_{k}} & y_{ij}=1\\ 0 & otherwise \end{array}$$

where *d* is the degree of the vertex, p is the prior distribution of the class and constrained to $\sum_{j=1}^{c}p_{j}=1$. In this paper, $p_{j}=1/c$ by default.

The final optimization issue is rewritten as follows:

(16) $$\begin{array}{l} Y^{*}=\arg\;\min\frac{1}{2}\mathrm{tr}\left(Y^{T}\Lambda A\Lambda Y\right)\\ \;s.t.\;y_{ij}\in \{0,1\},\\ \;\sum_{j=1}^{c}y_{ij}=1,j=1,\cdots ,c.\\ \;y_{ij}=1,for\;\mathrm{label}\left(x_{i}\right)=j,j=1,\cdots ,c. \end{array}$$

The optimization is a NP hard problem which requires solving a linearly constrained binary integer programming(BIP) problem [32]. A forthright way to work out the minimization problem is to update the label variable Y with the gradient descent method. Wang and Jebara [16] has proved that the minimization problem equals to a Max K-Cut problem (K = c) over the graph $G_{A}=\left\{X,A\right\}$.

Therefore, this study utilizes a greedy gradient Max-Cut algorithm to find local optima by selecting unlabeled vertices randomly and placing each of them into the appropriate class subset with minimum connectivity to maximize cross-set edge weights iteratively. The connectivity between unlabeled vertex xi and labeled subset S are defined as follows:

(17) $$c_{ij}=p_{j}\cdot \sum_{m=1}^{n}\lambda_{m}a_{im}y_{mj}=p_{j}\cdot \Lambda A_{i\cdot }Y_{\cdot j}$$

(18) $$S_{j}=\left\{x_{i}|y_{ij}=1\right\},i=1,2,\cdots ,n;j=1,2,\cdots ,c$$

The greedy gradient Max-Cut Algorithm 1.

**Algorithm 1.** Greedy Gradient Max-Cut
1**Input**: the graph $G_{A}=\left\{X,A\right\}$ and labeled vertex $X_{l}$ and label Y;2**Initialization**:3Construct the initial cut $\left\{S_{j}\right\}$ by the initial labeled vertex $X_{l}$:$S_{j}=\left\{x_{i}|y_{ij}=1\right\},j=1,2,\cdots ,c$4Unlabeled vertex set $X_{u}=XX_{l}$;5**repeat**6    for all j=0 to $\left|X_{u}\right|$ **do**7         calculate the connectivity:$c_{ij}=\sum_{k=1}^{n}\lambda_{k}a_{ik}y_{kj},x_{i}\in X_{u},j=1,\cdots ,c$8    **End for**9    Update the cut $\left\{S_{j}\right\}$ by placing the vertex xi* to the Sj* subset:$\left(i^{*},j^{*}\right)=\arg\underset{i,j,x_{i}\in X_{u}}{\min}c_{ij}$10    Add xi to $X_{l}:X_{l}\leftarrow X_{l}+x_{i}；$11Delete xi from $X_{u}:X_{u}\leftarrow X_{u}-x_{i};$12**Until** $X_{u}=\emptyset$13**Output**: the final cut and the corresponding labeled subsets $S_{j},j=1,2,\ldots ,c$Sj, j = 1, 2,…, *c*


## 4. Simulation Experiment for VGF

To demonstrate the performance of VGF, a faulty simulated rolling bearing vibration signal with different resonant frequency under variable speeds is generated and analyzed. The simulated signal can be generated as follows [33]:

(19) $$x\left(t\right)=\sum_{k=-N}^{N}Ae^{-\alpha \left(t-k*p*f_{r}-\sum_{i=-N}^{k}\tau_{i}\right)}\sin\left(2\pi f_{m}\left(t-k*p*f_{r}-\sum_{i=-N}^{k}\tau_{i}\right)\right)u\left(t-k*p*f_{r}-\sum_{i=-N}^{k}\tau_{i}\right)+n\left(t\right)$$

where, α = 0.03 is the structural damping characteristic, $f_{r}$ = *n_s_*/60 (*n_s_* means the shaft speed) is the shaft rotation frequency, $f_{m}$ = 2000 Hz is the resonant frequency, $A=f_{r}/20$ is the amplitude of the impulse. *p* represents the number of fault impulses in every shaft revolution (here *p* = 3.58). *τ_i_* denotes the randomness of rolling elements slippage, which is subject to a uniformly distribution with a zero mean and a standard deviation of $\left(0.01\sim 0.02\right)f_{r}$. n(t) is a white Gaussian noise with a signal-to-noise ratio of 0 dB.

**Figure 5:**
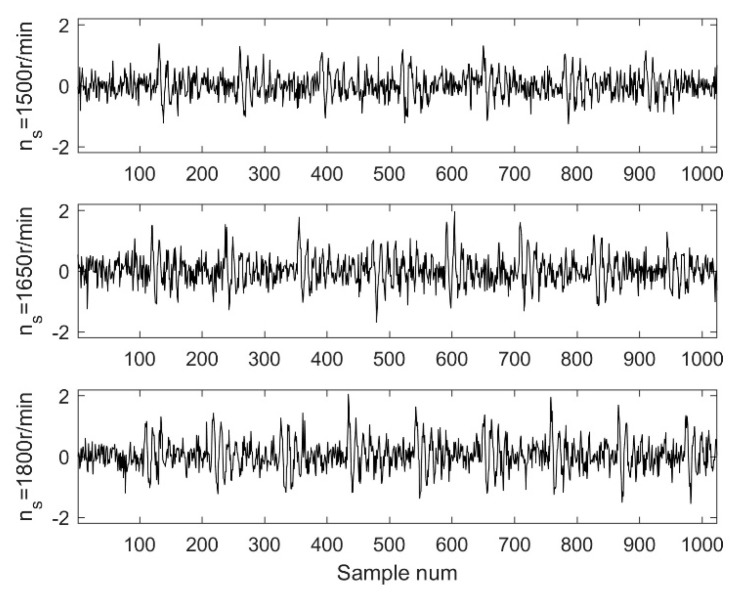
The simulated signal with different *n_s._*

To simulate variable conditions, this study assumed that the shaft speed varies from 1500 to 1800 r/min, and the step size defaults to 30 r/min. Therefore, 11 groups of simulated signals were gathered with a sample frequency of 12 kHz, as shown in Figure 5.

To verify the VGF, the signals were segmented into samples with a fixed length (*N* = 1024). Then, GIC was extracted from the samples. For comparisons, Approximate Entropy [34], Sample Entropy [35], and Fuzzy Entropy [36] are involved in this study. This paper set the embedding dimension *m* = 2, similarity criterion *r* = 0.15 of the standard deviation of a signal, length of data *N* = 1024, and fuzzy power *n* = 2.

The averages of features are calculated. There are 11 values corresponding to different speeds for each method, as shown in Table 1. It is obvious that GIC has the smallest standard deviation which means it is natively insensitive to variable conditions.

## 5. Experimental Analysis

### 5.1. Experimental Setup

**Figure 6:**
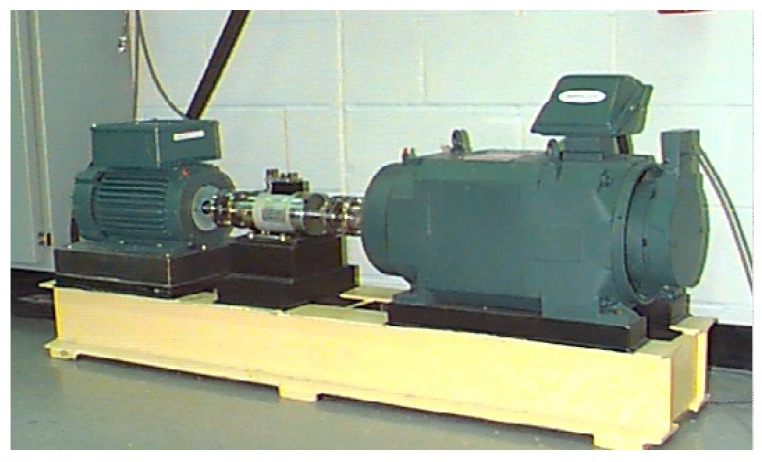
Test-rig of the rolling bearing [37].

This study utilized the data collected by the Case Western Reserve University Bearing Data Center to verify the proposed method. The test rig is shown in Figure 6, the shaft is driven by a 2 hp (1470 W) reliance electric motor, a torque transducer, and an encoder mounted on the shaft (Details about the test rig and experimental data can be found in [37].). The tested bearings are 6205-2RS JEM SKF, deep groove ball bearings. The faults were introduced using electro-discharge machining ranging from 0.007 inches (0.178 mm) in diameter to 0.040 inches (0.355 mm) in diameter separately at the inner race, rolling element, and outer race. The location of faults at the fixed outer race was considered with 3 o’clock, 6 o’clock and 12 o’clock. With the motor loads ranging from 0 to 3 horsepower (0 to 2205 W) (motor speeds of 1797 to 1720 r/min), vibration data was recorded at sample frequencies of 12 kHz and 48 kHz.

To demonstrate the superiority of the proposed VGF-GRSSL method, this study randomly selected a set of imbalanced data with unlabeled samples under variable conditions for comparison and analysis. The dataset under the defect of 0.007 inches (0.178 mm) and sample frequencies of 12 kHz is showed in the Table 2. The vibration data was divided into four types including normal, inner race fault, outer race fault, and rolling element fault. All of them consist of four operating conditions with the load range from 0 to 3 horsepower (0 to 2205 W) and speed range from 1797 to 1720 r/min, respectively. Each sample contains 1024 points. Therefore, there are 3081 samples (1657 for normal, 476 for inner fault, 475 for outer fault, and 473 for element fault). Generally speaking, the sampling time for each data sample is extremely short. For example, when the sample frequency is set to 12 kHz and the length of each data sample *N* = 1024, the sampling time is less than 0.085 s. During such a short time period, the operation condition is considered as constant. Therefore, the four different operation conditions of the Case Western data can be considered under variable conditions.

### 5.2. Fault Diagnosis Based on VGF-GRSSL

**Figure 7:**
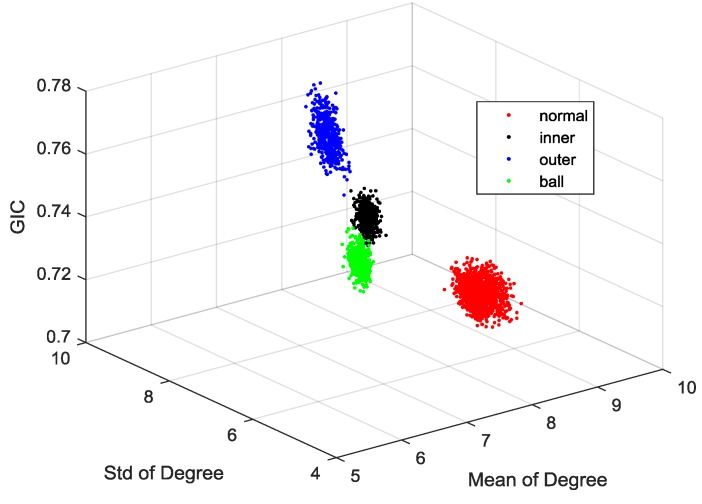
Feature space.

The VGFs were extracted, as showed in the Figure 7. It is observed that the locations of features are separated according to their fault modes, remarkably. Therefore, the VGF method not only can extract fault features, but also has strong robustness to variable conditions.

Based on the obtained features, the GRSSL method is employed for fault classification. For comparisons, GFHF and LGC are involved in this study. This paper set the hyper-parameter µ = 0.01 for LGC and GRSSL. The algorithms GRSSL, GFHF, and LGC shared the same graph construction procedure. The standard k-nearest-neighbors (set *k* = 6) method was employed for the sparsification and Gaussian kernel weighting for reweighting the edges. In the Gaussian kernel, this study used the ℓ2 distance, and the kernel bandwidth σ is defined as the average distance between each selected sample and its k’th nearest neighbor.

In the case of the multi-class problem, the imbalanced ratio is defined as the number of majority samples divided by the sum of number of minority samples. It is assumed that the number of each minority class contains the same number of samples.

To demonstrate the performance of the proposed method under different imbalanced ratios, for the labeled data, we fix the minority classes (including inner fault, outer fault, rolling element fault) to have only three label samples and then randomly select *m* labels from the majority class (normal). For the unlabeled data, we keep it with the same imbalanced ratio as the labeled data. Here, we varied the ratio (*r* = *m*/3) from 1 to 20.

**Figure 8:**
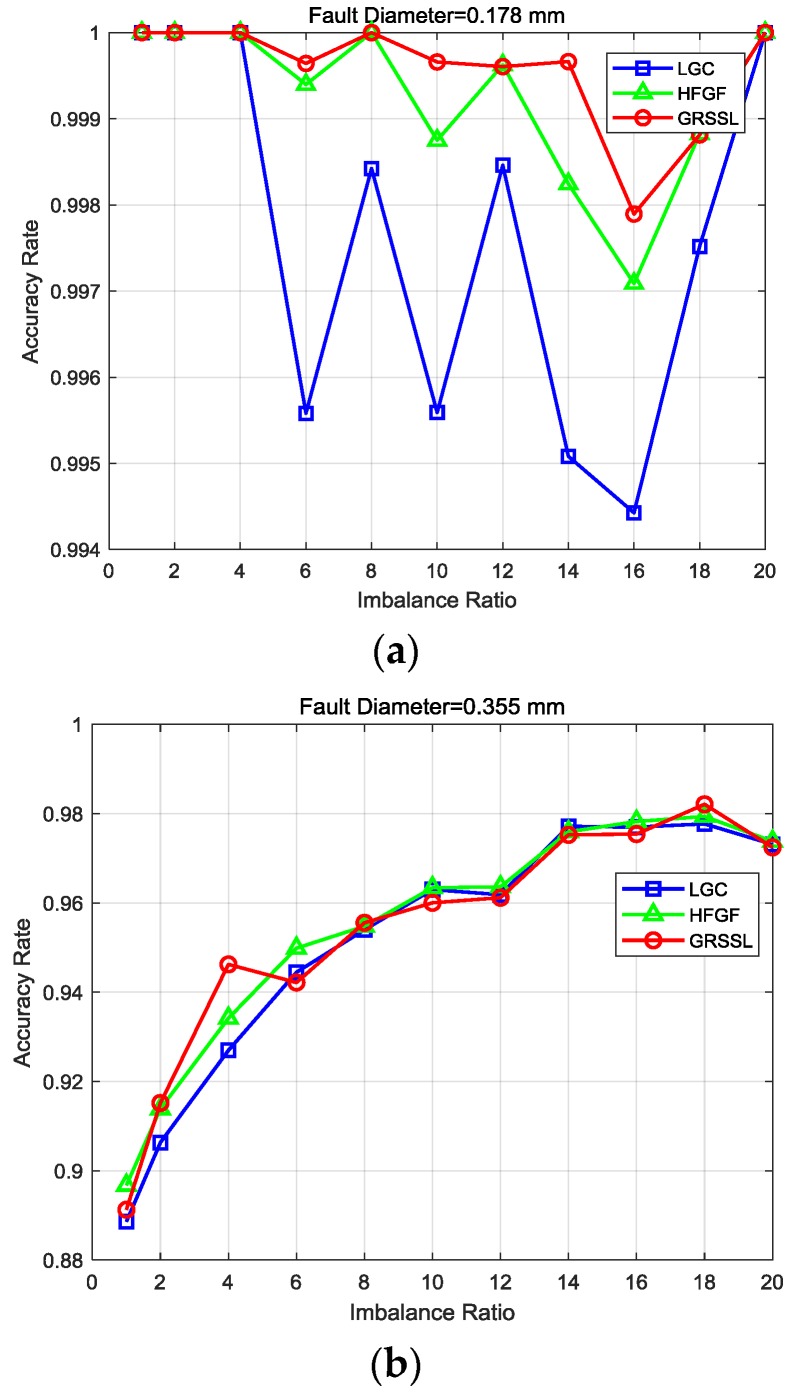

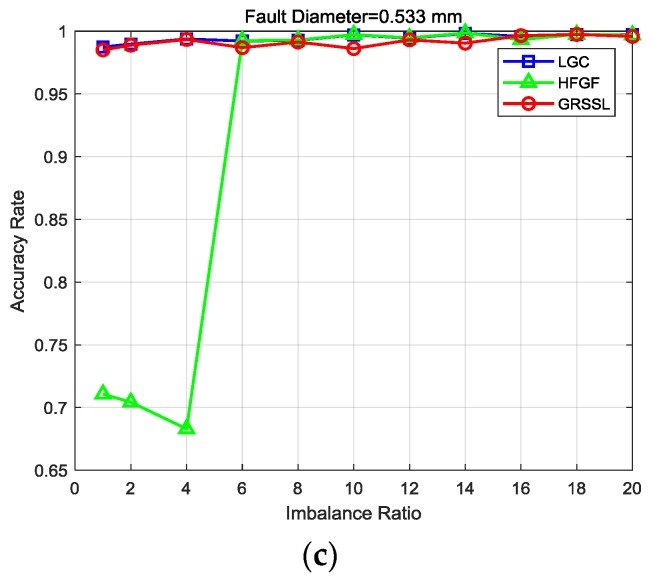
Performance of Local and Global Consistency (LGC), Gaussian Fields and Harmonic Functions (GFHF), and graph-based rebalance semi-supervised learning (GRSSL) algorithms using the bearing data with a fault diameter of (**a**) 0.178 mm, (**b**) 0.355 mm and (**c**) 0.533 mm.

The results are shown in Figure 8 under three conditions (i.e., fault diameter 0.007, 0.014, and 0.021 inches (0.178 mm, 0.355 mm, and 0.533 mm)). In Figure 8a, it can be observed: (1) all three algorithms can obtain accuracy rates of nearly 100% when the imbalance ratio is less than 4, and (2) the accuracy of the GRSSL is more stable than the LGC and GFHF when the fault diameter is 0.007 inches (0.178 mm). Figure 8b illustrates results with a fault diameter of 0.014 inches (0.355 mm). The three methods obtained similar accuracy rates with varying imbalance ratios. Meanwhile, it is shown in Figure 8c that all methods achieved excellent accuracy rates except the GFHF with the imbalance ratio lower than 4. In summary, the GRSSL achieves a more stable accuracy rate than the LGC and GFHF under variable conditions with imbalanced unlabeled data.

To illustrate the diagnostic performance of the proposed method exactly, we calculated the accuracy of each class, which is displayed in the Table 3. It is evident that the GRSSL method outperformed other methods under condition 1 and condition 3. However, all methods got a bad performance for the inner race fault and ball fault under the condition 2. But they recognized the normal samples and faulty samples at rate of 100%, which is of significance to industrial field application.

## 6. Conclusions

Rolling element bearings are vital to rotating machineries. This paper proposes a novel hybrid method combined with visibility graph feature (VGF) and graph-based rebalance semi-supervised learning (GRSSL) for bearing fault diagnosis under variable conditions with imbalanced unlabeled data. Firstly, the visibility graph algorithm is used to extract features which are natively insensitive to variable conditions. Secondly, the GRSSL is utilized to make effective use of the imbalanced unlabeled data for fault classification. Experiment results have demonstrated the superiority of the proposed VGF and GRSSL: (1) Compared with approximate entropy, sample entropy, and fuzzy entropy, the VGF can effectively extract the fault characteristics under variable conditions; (2) GRSSL has superior performance in bearing fault diagnosis under variable conditions with imbalanced unlabeled data.

However, to some extent, the imbalanced ratio involved in this study only considered normal samples and faulty samples. The absence of faulty samples, such as inner race fault and outer race fault, may create new problems. Therefore, additional experiments under more different ratios should be done to validate and improve the method. Meanwhile, more attention should be paid to the development of semi-supervised learning diagnosis methods.

## Figures and Tables

**Table 1:** Features of the simulated signal.

Shaft Speed/Feature	GIC	ApEn	SampEn	FuzEn
1500 r/min	0.046	1.485	2.288	0.782
1530 r/min	0.046	1.496	2.313	0.780
1560 r/min	0.046	1.485	2.272	0.776
1590 r/min	0.047	1.496	2.319	0.795
1620 r/min	0.046	1.478	2.268	0.783
1650 r/min	0.047	1.478	2.252	0.786
1680 r/min	0.045	1.472	2.240	0.774
1710 r/min	0.047	1.469	2.209	0.773
1740 r/min	0.047	1.476	2.243	0.786
1770 r/min	0.046	1.458	2.197	0.782
1800 r/min	0.046	1.460	2.184	0.762
Std of features	0.0005	0.0126	0.0444	0.0085

**Table 2:** Dataset of 0.007 inches (0.178 mm) defect.

Dataset	Fault Type	Operating Condition	Motor Load (hp)	Sample Num
1	Normal	1797 r/min, 0 HP	0 (0 W)	238
		1772 r/min, 1 HP	1 (735 W)	472
		1750 r/min, 2 HP	2 (1470 W)	473
		1730 r/min, 3 HP	3 (2205 W)	474
2	Inner race fault	1797 r/min, 0 HP	0 (0 W)	118
		1772 r/min, 1 HP	1 (735 W)	119
		1750 r/min, 2 HP	2 (1470 W)	119
		1730 r/min, 3 HP	3 (2205 W)	120
3	Outer race fault	1797 r/min, 0 HP	0 (0 W)	119
		1772 r/min, 1 HP	1 (735 W)	119
		1750 r/min, 2 HP	2 (1470 W)	118
		1730 r/min, 3 HP	3 (2205 W)	119
4	Rolling element fault	1797 r/min, 0 HP	0 (0 W)	119
		1772 r/min, 1 HP	1 (735 W)	118
		1750 r/min, 2 HP	2 (1470 W)	118
		1730 r/min, 3 HP	3 (2205 W)	118

**Table 3:** Results of LGC, GFHF, and GRSSL under different severity of fault with the imbalance ratio *r* = 16 (IRF–inner race fault, ORF-outer race fault, BF-ball Fault).

Fault Diameter	Method	Classification Accuracy (%)				Average Accuracy (%)
		Normal	IRF	ORF	BF	
Condition 1 (0.178 mm)	GRSSL	100.00	**100.00**	**99.06**	96.92	**99.79**
	LGC	100.00	94.46	97.84	97.06	99.44
	GFHF	100.00	97.72	99.04	97.68	99.71
Condition 2 (0.355 mm)	GRSSL	100.00	77.84	96.82	62.94	96.73
	LGC	100.00	84.56	98.54	71.24	97.61
	GFHF	100.00	82.64	98.78	71.92	97.56
Condition 3 (0.533 mm)	GRSSL	100.00	**100.00**	**98.06**	95.12	**99.64**
	LGC	100.00	97.48	96.04	96.08	99.46
	GFHF	100.00	98.68	97.06	95.68	99.55
